# Changes in the metabolomic profiles of mammary secretion in relation to dam litter size and parity number in Black Bengal goats

**DOI:** 10.14202/vetworld.2024.1469-1481

**Published:** 2024-07-07

**Authors:** Tien Thi Phuong Vo, Chollada Buranakarl, Sumonwan Chamsuwan, Sumpun Thammacharoen, Panupat Ratchakom, Morakot Nuntapaitoon, Sarn Settachaimongkon

**Affiliations:** 1 Department of Physiology, Faculty of Veterinary Science, Chulalongkorn University, Bangkok, Thailand; 2 Department of Livestock Development, Chiang Rai Provincial Livestock Office, Chiang Rai, Thailand; 3 Department of Obstetrics, Gynaecology and Reproduction, Faculty of Veterinary Science, Chulalongkorn University, Bangkok, Thailand; 4 Multi-Omics for Functional Products in Food, Cosmetics and Animals Research Unit, Chulalongkorn University, Bangkok, Thailand; 5 Department of Food Technology, Faculty of Science, Chulalongkorn University, Bangkok, Thailand

**Keywords:** black Bengal goats, colostrum, litter size, metabolite, milk, parity number

## Abstract

**Background and Aim:**

The colostrum is essential for a kid’s survival and development. The metabolomic profiles of mammary secretion in goats are limited. This study investigated the metabolomic profiles of mammary secretion in purebred Black Bengal goats and their relationships with litter size and parity number.

**Materials and Methods:**

500 MHz nuclear magnetic resonance was used to analyze the metabolomic profiles of 43 colostrum and milk samples collected on delivery day and day 7 after parturition, respectively.

**Results:**

Fifty-one metabolites were distinguished between colostrum and milk based on heatmap visualization and hierarchical cluster analysis. In colostrum, most compounds were present in significantly greater amounts than in milk. Milk of goats with multiple litter sizes had higher levels of lactose while fat, protein, total solids, solid not fat, and most of the metabolites were lower. The parity number of dams shows no difference in the composition of all components between primiparous and multiparous goats.

**Conclusion:**

The components in colostrum were significantly more concentrated than those in milk. The multiple litter sizes in dams led to a significant impact on the composition of lactose and other milk metabolites.

## Introduction

The goat population has grown significantly due to its high nutritional value and human health benefits. The small-size Black Bengal (BB) goat is cultivated extensively for meat and skin due to its resilience to harmful conditions and excellent qualities in Asian lands [1]. Newborn BB kids with low milk production in high litter sizes face increased mortality risk [2].

After delivery, the colostrum is the earliest mammary secretion rich in nutrients, growth factors, and passive antibodies. Colostrum is gradually replaced by mature milk. Adequate colostrum supply as the primary source of nutrition significantly impacts neonatal survival [3]. The colostrum’s insulin-like growth factor 1, immunoglobulin G, and Vitamin A (retinol) content have been linked to improved neonatal growth [4]. The nutritional composition of mammary secretion, including its major constituents such as fat, proteins, lactose, growth factors, and immune-related compounds, can be significantly influenced by nutrition, herd management, and environmental conditions. Colostrum content was influenced by reported litter size and parity number [5, 6]. The presence and variation of other minor compounds in mammary secretion remain an unexplored research area.

Goat colostrum studies have primarily focused on macromolecule compositions. Metabolomics, involving the analysis of small-molecule-weight metabolites (<1.5 kDa) in mammary secretion and other bodily fluids for lactation and dairy research, has gained recognition recently [7, 8]. Using the metabolomics approach can assist in a comprehensive understanding of metabolic pathways in the animal body and the characteristics of milk and dairy pro-ducts. Recently, proton nuclear magnetic resonance (^1^H-NMR) has been used as one of the most reliable techniques for determining the characteristics of dairy ruminant milk through metabolomic profile investigation [9]. ^1^H nuclei produce a magnetic resonance signal by behaving as electrically charged spins in a magnetic field [10]. ^1^H-NMR studies have revealed metabolomic changes in mammary secretion during transition in multiple species [11, 12]. Goat milk metabolomic analysis has been utilized for examining different breeds [13], linking to animal health and heat stress adaptation [14–16].

In the peripartum period, the dam’s metabolic needs peak while essential nutrients are intensively secreted by the mammary glands to the developing offspring. Metabolomic changes during the peripartum period mirror both dam health and kid’s growth development. The effect of dam’s litter size and parity number on goat milk profile remains limited.

This study aimed to examine metabolic variation between colostrum and milk in BB goats, depending on dam litter size and parity number, under tropical conditions in northern Thailand.

## Materials and Methods

### Ethical approval

The study was conducted according to the Animal Care and Use Protocol approved by the Animal Care and Use Committee, Faculty of Veterinary Science, Chulalongkorn University (Protocol number: 2231025).

### Study period and location

The study was conducted from March to October-2022 The metabolomic profiles were investigated from mammary secretions from 43 dams at the Chaipattana Foundation’s Black Bengal Goat Domestication Project Farm, located in Chiang Rai Province, northern Thailand.

### General management

Forty-three purebred BB goats were used in this study. The average maximal temperature and minimal temperature during the day were 32.5 ± 0.2°C and 21.9 ± 0.1°C, respectively, while the relative humidity was 80.8 ± 0.4% (range 63%–95%).

Most females older than 6 years old were not used for breeding. The pregnant dams, regardless of parity number from 1 to 7, were reared in a conventional opened-housing system with 2–3 goats in each pen (3 × 3 m). After parturition, the dam was moved to individual pens with her kids until weaning. The adult goats were fed commercial concentrate of approximately 200 g/goat per day and roughage, including 2 kg/goat per day of Napier grass and 1 kg/goat per day of Pangola hay (feedstuff chemical compositions are presented in Table-1). All goats were fed twice a day at 07:00 and 18:00 h according to the standard management protocol with free water access. All dams were allowed to graze for approximately 3–4 h/day, depending on the weather conditions. During the rainy season, goats were limited to grazing outside.

**Table-1:** The chemical compositions of goat diet (% DM basis).

Composition	Concentrate	Napier grass	Pangola hay
Dry matter	91.15	16.22	92.77
Organic matter	85.73	88.72	94.58
Ash	14.27	11.28	5.42
Crude protein	17.24	15.04	3.54
Crude fat	2.92	1.85	1.21
Crude fiber	12.58	30.76	30.59
NDF	34.06	61.20	66.24
ADF	16.35	39.30	37.58

DM=Dry matter, NDF=Neutral detergent fiber, ADF=Acid detergent fiber

Regular anthelmintic medication and pesticide treatments (dipping/spraying) were administered to monitor and control parasites. Annually, all goats received vaccination against foot-and-mouth disease virus. Routine blood tests were performed to rule out caprine arthritis, encephalitis, and brucellosis. Dams were naturally mated with chosen BB bucks. Advanced pregnancy dams were housed in separate pens for superior peripartum care.

### Sample collection

50 mL of colostrum and milk were manually obtained from each dam using standard techniques. Colostrum was gathered within 3 h postpartum (D0), while milk sampling took place between 8:00 and 10:00 am on day 7 (D7). The samples were stored at –20°C in disposable plastic tubes until the meta- bolome and composition analysis, completed within 3 months post-collection.

### Analytical procedure

#### Major chemical compositions

Approximately 20 mL of colostrum and milk were thawed by incubation in a water bath set at 40°C for 20 min. The major chemical compositions including fat, protein, lactose, solid not fat (SNF), and total solids (TS) of samples were determined using a MilkoScan FT2 instrument (Foss Milkoscan, Hillerøed, Denmark).

#### Sample preparation and metabolite analysis using ^1^H-NMR

Non-volatile polar metabolite compositions in colostrum and milk were analyzed according to the methods applied with some modifications [14, 17]. In brief, the pH of the defrosted samples was adjusted to 6.0 using 1.0 N HCl or 1.0 N sodium hydroxide. Colostrum and milk samples were diluted with ultrapure water at a ratio of 1:2 (v/v) and 1:1 (v/v), respectively. The samples were then centrifuged at 3000× *g* for 50 min at 10°C (Sorvall Legend XTR Centrifuge, Thermo Fisher Scientific, Massachusetts, USA). The skim milk fraction was collected and added to dichloromethane (RCI Labscan Ltd, Bangkok, Thailand) at a ratio of 1:2 (v/v). Samples were vigorously vortexed for 30 s and centrifuged at 5000× *g* for 40 min at 4°C to eliminate the lipid fraction. Large protein parts were removed from the skim milk by ultracentrifugation at 74,500× *g* for 60 min at 4°C (Optima™ L100-XP ultracentrifuge, Beckman Colter, Inc., CA, USA). The clear milk serum was collected and filtered through a 3-kDa molecular weight cut-off Pall Nanosep^®^ centrifuge device (Pall Life Sciences, Ann Arbor, MI, USA) applied in combination with a centrifugation at 13,800× *g* for 20 min at ambient temperature. Three hundred μL of filtrate was collected and added to phosphate buffer pH 6.0, consisting of 300 mM KH_2_PO_4_ (Merck, Darmstadt, Germany), 10% (w/w) D_2_O (Cambridge Isotope Laboratories, Inc., Tewksbury, MA, USA), and 1 mM 3-(Trimethylsilyl) propionic-2, 2, 3, 3-d4 acid sodium salt (TSP) (Merck, Darmstadt, Germany) as an internal standard at a ratio of 1:1 (v/v). Finally, 400 μL of the mixture was supplemented with 200 μL of D_2_O and then subjected to a Bruker Avance III HD 500 MHz NMR spectrometer (Bruker, Rheinstetten, Germany) operated under full automation.

#### ^1^H-NMR spectral processing and data acquisition

^1^H-NMR spectra of samples were phase corrected, baseline corrected, and normalized against the TSP internal standard. Chemical shifts ranging from 0.00 to 10.00 were segmented with 0.02 ppm intervals and integrated by binning in Bruker TopSpin 4.2.0 software (Bruker BioSpin, Rheinstetten, Germany). Identification of metabolites present in colostrum and milk samples was performed using the Chenomx NMR suite 8.6 library (Chenomx Inc., Edmonton, Alberta, Canada), Milk Composition Database (www.mcdb.ca), and Livestock Metabolome database (www.lmdb.ca). The relative quantifications of metabolites were normalized and adjusted to log10 and log2 scales before univariate and multivariate statistical analyses, respectively.

### Statistical analysis

Descriptive characteristics of dams are presented as mean ± SE, minimum, and maximum values using SAS 9.4 (SAS Institute, Inc., Cary, NC, USA). The major chemical and metabolite compositions of colostrum and milk are shown as mean ± SE and compared using the Student Paired t-test. The effects of parity number and litter size on mammary secretion and major chemical and metabolite compositions were analyzed using a general linear model procedure. The fixed effects in the model included litter size (single and multiple) and parity number (primiparous and multiparous). Data are presented as least square mean ± standard error of the mean.

Metabolomic profiles of samples were analyzed and compared with chemometrics using the MetaboAnalyst 6.0 online software platform (www.metaboanalyst.ca). Heatmap visualization combined with Pearson’s correlation-based hierarchical clustering was applied for the overall comparison of sample metabolite profiles. Partial least square discriminant analysis (PLS-DA) was performed to evaluate the effect of litter size and parity number on the metabolite profiles of colostrum and milk. The quality of the PLS-DA model was expressed by *R*^2^ (accuracy) and *Q*^2^ values (predictability) derived from a leave-one-out cross-validation test. Finally, metabolites with variable importance in projection (VIP) score >1.0 and p ≤ 0.05 were considered as potential biomarkers responsible for discrimination.

## Results

### Descriptive statistics of experimental dam characteristics

Forty-three dams in this study had an average age of 29.4 ± 2.5 months (8–70 months). The average parity number was 2.63 ± 0.27 (1–7), while litter size was 1.79 ± 0.11 (1–4). The numbers of dams with first, second, third, and fourth kids per litter were 16, 21, 5, and 1, respectively.

### Overall variations in the major chemical composition and metabolite profiles of colostrum and milk

When colostrum transitioned into mature milk on day 7, the concentrations of fat and protein dramatically decreased (20.0% and 71.8%, respectively), whereas lactose concentration significantly increased, resulting in lower TS and SNF (Table-2).

**Table-2:** Quantification of major chemical composition and non-volatile polar metabolites identified in colostrum and milk of black Bengal goats.

Parameters	Chemical shift*	Concentrations	

		Colostrum	Milk
Chemical composition, %			
Fat		9.13 ± 0.47	6.570 ± 0.273***
Protein		15.67 ± 0.69	4.42 ± 0.21***
Lactose		2.99 ± 0.11	4.58 ± 0.05***
TS		29.14 ± 0.84	16.92 ± 0.36***
SNF		20.73 ± 0.62	10.38 ± 0.16***
Metabolomic profiles^1^			
Amino acids and derivatives			
Alanine	1.48 (d), 3.79 (m)	7.14 ± 0.10	6.40 ± 0.09***
Betaine	3.28 (s), 3.91 (s)	7.99 ± 0.03	8.18 ± 0.02***
Creatine	3.04 (s), 3.92 (s)	7.26 ± 0.06	6.87 ± 0.05***
Creatine phosphate	3.04 (s), 3.97 (s)	7.26 ± 0.06	6.87 ± 0.05***
Creatinine	3.04 (s), 4.06 (s)	7.60 ± 0.04	7.21 ± 0.04***
Glycine	3.57 (s)	8.12 ± 0.03	8.30 ± 0.02***
Isoleucine	0.93 (d), 1.01 (d), 3.69 (d)	7.85 ± 0.11	6.87 ± 0.12***
Leucine	0.95 (d), 0.97 (d), 3.73 (m)	7.73 ± 0.11	6.71 ± 0.13***
Methionine	2.14 (s), 3.86 (m)	7.19 ± 0.08	6.67 ± 0.07***
Proline	2.06 (m), 3.43 (m)	7.90 ± 0.07	7.24 ± 0.07***
Sarcosine	2.73 (s), 3.61 (s)	6.58 ± 0.07	6.12 ± 0.07***
Threonine	1.33 (d), 3.62 (d)	7.33 ± 0.09	6.49 ± 0.09***
Tyrosine	3.21 (m), 3.96 (m)	8.32 ± 0.03	7.82 ± 0.03***
Valine	0.99 (d), 1.05 (d), 3.63 (d)	7.67 ± 0.10	6.58 ± 0.12***
Organic acids and derivatives			
3-hydroxybutyrate	1.23 (d)	7.63 ± 0.09	6.85 ± 0.52***
Acetate	1.93 (s)	6.92 ± 0.11	5.95 ± 0.11***
Butyrate	0.90 (t), 1.56 (m), 2.17 (t)	7.91 ± 0.11	7.17 ± 0.10***
Citrate	2.56 (d), 2.70 (d)	7.97 ± 0.04	7.88 ± 0.04
Fumarate	6.52 (s)	6.54 ± 0.04	5.99 ± 0.05***
Hippurate	7.56 (t), 7.84 (d)	7.07 ± 0.04	6.60 ± 0.04***
Lactate	1.33 (d), 4.12 (m)	7.33 ± 0.09	6.49 ± 0.09***
Malonate	3.15 (s)	6.43 ± 0.07	6.29 ± 0.04
Pyruvate	2.38 (s)	7.06 ± 0.10	6.42 ± 0.08***
Succinate	2.41 (s)	6.65 ± 0.10	6.20 ± 0.06***
Threonate	4.03 (d)	7.07 ± 0.04	6.80 ± 0.04***
Valerate	0.90 (t), 2.16 (t)	7.95 ± 0.10	7.22 ± 0.10***
Carbohydrate and derivatives			
Levoglucosan	3.54 (s), 4.08 (d), 5.47 (s)	8.08 ± 0.03	8.02 ± 0.03
Galactose	3.50 (m), 3.81 (m), 5.27 (d)	7.04 ± 0.24	6.62 ± 0.05***
Glucose	3.40 (t), 3.49 (t), 3.90 (m), 5.24 (d)	7.55 ± 0.04	7.37 ± 0.03***
Lactose	3.30 (t), 3.55 (m), 3.73 (m), 3.95 (m), 4.45 (d), 5.24 (d)	8.77 ± 0.03	8.92 ± 0.02***
Mannitol	3.68 (m), 3.81 (d), 3.86 (m)	8.76 ± 0.03	8.93 ± 0.02***
Myo-inositol	3.54 (m), 3.61 (t), 4.05 (t)	7.07 ± 0.04	6.76 ± 0.04***
N-acetylglucosamine	2.04 (s), 2.06 (s), 3.46 (m)	7.74 ± 0.07	7.00 ± 0.07***
UDP-galactose	4.04 (d), 5.64 (m), 5.97 (d), 7.96 (d)	7.71 ± 0.03	7.39 ± 0.03***
UDP-glucose	3.55 (m), 4.30 (s), 5.97 (d), 7.96 (d)	7.93 ± 0.04	7.55 ± 0.04***
UDP-N-acetylglucosamine	5.52 (m), 5.97 (d), 7.96 (d)	7.74 ± 0.04	7.21 ± 0.04***
Lipids and derivatives			
Acetyl-carnitine	2.14 (s), 3.2 (s), 5.59 (m)	7.63 ± 0.05	7.23 ± 0.05***
Carnitine	3.21 (s), 3.40 (d), 3.41 (t)	7.64 ± 0.03	7.46 ± 0.03***
Choline	3.20 (s), 3.52 (m)	7.39 ± 0.03	7.07 ± 0.04***
GPC	3.23 (s), 4.33 (m)	8.31 ± 0.03	7.80 ± 0.03***
PC	3.23 (s), 3.62 (m), 4.20 (m)	8.26 ± 0.03	7.72 ± 0.03***
Triox	3.28 (s)	8.09 ± 0.03	8.25 ± 0.02***
Vitamins			
Vitamin B2 (riboflavin)	2.58 (s)	7.41 ± 0.04	7.21 ± 0.05**
Vitamin B5 (pantothenate)	0.91 (s), 0.95 (s), 3.99 (s)	7.87 ± 0.12	6.93 ± 0.13***
Vitamin B7 (biotin)	1.74 (m), 3.30 (m)	7.66 ± 0.10	6.74 ± 0.11***
Vitamin C (ascorbic acid)	3.74 (m), 4.52 (d)	7.70 ± 0.03	7.34 ± 0.03***
Others			
Acetone	2.23 (s)	6.62 ± 0.10	5.81 ± 0.09***
Adenine	8.20 (s), 8.24 (s)	6.84 ± 0.07	6.21 ± 0.08***
Ethanol	1.20 (t), 3.67 (m)	7.29 ± 0.09	6.49 ± 0.09***
Hypoxanthine	8.20 (s), 8.22 (s)	6.68 ± 0.08	5.99 ± 0.08***
Uridine	4.23 (t), 5.90 (d), 7.88 (d)	7.01 ± 0.04	6.46 ± 0.05***

*: Metabolite signal was recorded with 3-(Trimethylsilyl) propionic-2, 2, 3, 3-d4 acid sodium salt (TSP) signal as internal standard at 0.00 ppm; (s): Singlet, (d): Doublet, (t): Triplet, (m): Multiple peak. Data of concentrations are presented as mean ± SEM.

** =p < 0.01,

*** =p < 0.001 compared between colostrum and milk using Student Paired t-test. UDP=Uridine diphosphate, TS=Total solids, SNF=Solid not fat

In this study, a total of 51 non-volatile polar metabolites, including amino acids, organic acids, carbohydrates, lipid derivatives, vitamins, and other miscellaneous compounds, were presumptively identified in the colostrum and milk of BB goats by a non-targeted ^1^H-NMR metabolomics approach (Table-2). The ^1^H-NMR chemical shift assignments of these compounds, along with their relative quantification and statistically significant levels, are presented in Table-2. Results demonstrated that the concentrations of most metabolites, except betaine, glycine, lactose, mannitol, and trimethylamine N-oxide (triox), were significantly higher in colostrum than in mature milk.

**Figure-1:**
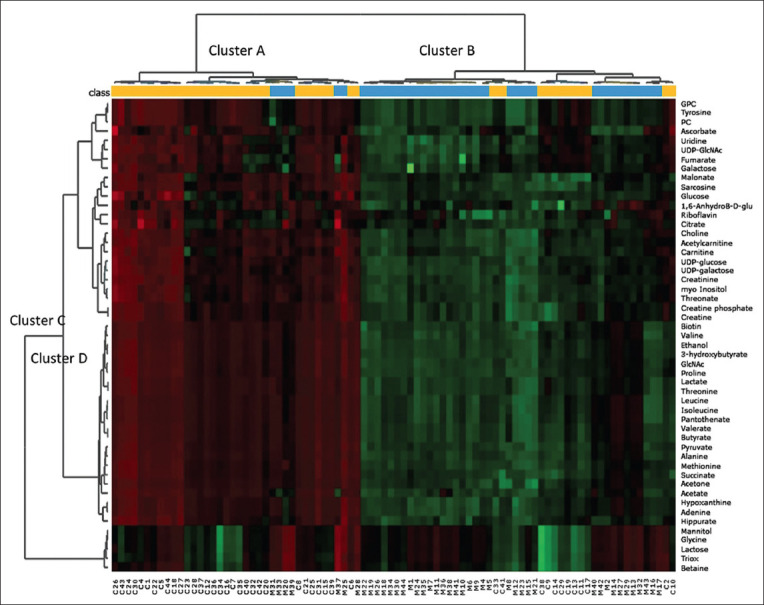
Heat-map visualization and hierarchical clustering of non-volatile polar metabolite profiles of colostrum (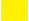) and mature milk (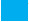) samples collected from purebred Black Bengal goats. Dendrogram represents sample clusters based on Pearson’s correlation coefficient with average linkage. Each square in the heat-map expresses normalized non-volatile polar metabolite content respected to the color range. The red color indicates higher content of the corresponding compound. For interpretation of the references to color in this figure, the reader is referred to the web version of this article or the relative quantification of metabolites in Table-2.

Heatmap visualization combined with hierarchical cluster analysis was performed to provide an overview of the differences in metabolite patterns among the samples (Figure-1). Results demonstrated a good distinction between colostrum (cluster A) and milk (cluster B) samples based on their ^1^H-NMR metabolite profiles. In addition, metabolites could be classified into different clusters based on the changes in their concentrations during the lactation period. Most compounds were present at significantly higher levels (red shading in heatmap) in colostrum than in milk (cluster C). Nevertheless, the concentrations of mannitol, glycine, lactose, triox, and betaine were found to be higher in milk than in colostrum (cluster D). This non-supervised pattern recognition corresponded well with the results presented in Table-2. Because the lactation period had a strong impact on the mammary secretion metabolome, the effects of litter size and parity number were further evaluated by a separate comparison of samples obtained from the same postpartum period, that is, colostrum (D0) and mature milk (D7).

### Effect of litter size on the major chemical composition and metabolite profiles of colostrum and milk

Litter size has minor effects on colostrum composition. Only colostrum fat and TS were significantly higher in multiple litters than in single litters (Table-3). However, litter size had strong effects on milk composition. Concentrations of fat, protein, TS, and SNF were statistically lower, whereas lactose was higher in multiple litters than in single litters.

**Table-3:** Effects of litter size on major chemical composition and non-volatile polar metabolites identified in colostrum and milk of Black Bengal goats.

Parameters	Colostrum		p-value	Milk		p-value
	
	Single (n = 16)	Multiple (n = 27)		Single (n = 16)	Multiple (n = 27)	
Chemical compositions, %						
Fat (g/100 g)	8.04 ± 0.76	10.12 ± 0.61*	0.048	7.45 ± 0.41	5.85 ± 0.33***	0.001
Protein (g/100 g)	14.05 ± 1.14	16.84 ± 0.92	0.077	5.23 ± 0.32	3.89 ± 0.26**	0.004
Lactose (g/100 g)	3.25 ± 0.18	2.82 ± 0.15	0.086	4.42 ± 0.09	4.69 ± 0.07*	0.027
TS (%)	26.66 ± 1.33	31.10 ± 1.08*	0.018	18.68 ± 0.52	15.82 ± 0.42***	<0.001
SNF (%)	19.43 ± 1.04	21.65 ± 0.85	0.123	10.92 ± 0.26	10.02 ± 0.21*	0.015
Metabolomic profiles^1^ Amino acids and derivatives						
Alanine	7.22 ± 0.16	7.15 ± 0.13	0.746	6.72 ± 0.13	6.21 ± 0.11**	0.006
Betaine	8.10 ± 0.04	7.93 ± 0.03*	0.005	8.22 ± 0.03	8.15 ± 0.03	0.137
Creatine	7.33 ± 0.09	7.23 ± 0.08	0.448	7.00 ± 0.08	6.78 ± 0.06*	0.043
Creatine phosphate	7.33 ± 0.09	7.23 ± 0.08	0.448	7.00 ± 0.08	6.78 ± 0.06*	0.043
Creatinine	7.33 ± 0.09	7.22 ± 0.08	0.448	7.30 ± 0.06	7.15 ± 0.05*	0.084
Glycine	8.22 ± 0.05	8.06 ± 0.04*	0.011	8.35 ± 0.03	8.27 ± 0.03	0.109
Isoleucine	7.93 ± 0.18	7.87 ± 0.15	0.822	7.39 ± 0.19	6.57 ± 0.15**	0.003
Leucine	7.81 ± 0.18	7.76 ± 0.15	0.837	7.24 ± 0.20	6.41 ± 0.16**	0.003
Methionine	7.26 ± 0.13	7.18 ± 0.10	0.642	6.85 ± 0.11	6.58 ± 0.09	0.066
Proline	7.96 ± 0.11	7.90 ± 0.09	0.658	7.52 ± 0.10	7.08 ± 0.08**	0.002
Sarcosine	6.68 ± 0.17	6.56 ± 0.14	0.606	6.28 ± 0.12	6.02 ± 0.10	0.109
Threonine	7.40 ± 0.14	7.33 ± 0.12	0.743	6.81 ± 0.14	6.32 ± 0.11*	0.011
Tyrosine	8.29 ± 0.05	8.34 ± 0.04	0.436	7.89 ± 0.05	7.77 ± 0.04	0.071
Valine	7.72 ± 0.17	7.68 ± 0.14	0.865	7.11 ± 0.17	6.28 ± 0.14***	<0.001
Organic acids and derivatives						
β-hydroxybutyrate	7.69 ± 0.14	7.64 ± 0.12	0.787	7.17 ± 0.12	6.67 ± 0.10**	0.004
Acetate	7.01 ± 0.16	6.92 ± 0.13	0.669	6.33 ± 0.13	5.72 ± 0.10***	<0.001
Butyrate	8.01 ± 0.18	7.92 ± 0.14	0.710	7.53 ± 0.16	6.97 ± 0.13*	0.010
Citrate	7.94 ± 0.07	7.98 ± 0.06	0.688	7.98 ± 0.06	7.87 ± 0.05	0.830
Fumarate	6.48 ± 0.07	6.60 ± 0.06	0.237	6.09 ± 0.09	5.94 ± 0.07	0.197
Hippurate	7.10 ± 0.07	7.07 ± 0.06	0.777	6.69 ± 0.07	6.55 ± 0.06	0.148
Lactate	7.40 ± 0.14	7.33 ± 0.12	0.743	6.81 ± 0.17	6.32 ± 0.11*	0.011
Malonate	6.57 ± 0.12	6.38 ± 0.10	0.243	6.42 ± 0.07	6.21 ± 0.06*	0.028
Pyruvate	7.09 ± 0.06	7.07 ± 0.05	0.849	6.73 ± 0.13	6.23 ± 0.10**	0.007
Succinate	7.15 ± 0.16	7.05 ± 0.13	0.645	6.41 ± 0.10	6.08 ± 0.08*	0.017
Threonate	6.76 ± 0.16	6.63 ± 0.13	0.565	6.89 ± 0.06	6.74 ± 0.05	0.059
Valerate	8.04 ± 0.17	7.95 ± 0.14	0.692	7.57 ± 0.15	7.02 ± 0.12*	0.011
Carbohydrate and derivatives						
Levoglucosan	7.93 ± 0.07	7.93 ± 0.06	0.951	8.11 ± 0.04	7.97 ± 0.03*	0.019
Galactose	6.98 ± 0.06	7.08 ± 0.05	0.249	6.74 ± 0.08	6.56 ± 0.06	0.099
Glucose	7.59 ± 0.06	7.53 ± 0.05	0.420	7.42 ± 0.04	7.33 ± 0.04	0.143
Mannitol	8.86 ± 0.05	8.70 ± 0.04	0.014	8.98 ± 0.03	8.90 ± 0.03	0.087
Myo-inositol	8.30 ± 0.04	8.16 ± 0.04*	0.025	6.86 ± 0.06	6.70 ± 0.05	0.061
Acetylglucosamine	7.78 ± 0.11	7.75 ± 0.09	0.850	7.33 ± 0.11	6.81 ± 0.09**	0.001
UDP-galactose	7.43 ± 0.06	7.44 ± 0.05	0.973	7.49 ± 0.05	7.33 ± 0.04*	0.034
UDP-glucose	7.24 ± 0.07	7.18 ± 0.05	0.525	7.65 ± 0.06	7.50 ± 0.05	0.065
UDP-acetylglucosamine	7.60 ± 0.06	7.61 ± 0.05	0.932	7.33 ± 0.06	7.15 ± 0.05*	0.024
Lipids and derivatives						
Acetylcarnitine	7.66 ± 0.08	7.63 ± 0.06	0.809	7.34 ± 0.08	7.17 ± 0.06	0.117
Carnitine	7.67 ± 0.05	7.64 ± 0.04	0.665	7.51 ± 0.05	7.42 ± 0.04	0.160
Choline	7.39 ± 0.06	7.39 ± 0.05	0.992	7.14 ± 0.07	7.03 ± 0.05	0.210
GPC	8.28 ± 0.05	8.34 ± 0.04	0.434	7.87 ± 0.04	7.76 ± 0.04	0.063
PC	8.23 ± 0.05	8.29 ± 0.04	0.402	7.78 ± 0.05	7.68 ± 0.04	0.118
Triox	8.18 ± 0.04	8.03 ± 0.03**	0.008	8.29 ± 0.03	8.22 ± 0.03	0.159
Vitamins						
Vitamin B2 (riboflavin)	7.39 ± 0.07	7.47 ± 0.06	0.447	7.24 ± 0.08	7.20 ± 0.07	0.688
Vitamin B5 (pantothenate)	7.95 ± 0.19	7.89 ± 0.15	0.807	7.43 ± 0.19	6.66 ± 0.16**	0.005
Vitamin B7 (biotin)	7.74 ± 0.16	7.67 ± 0.13	0.772	7.20 ± 0.16	6.48 ± 0.13**	0.002
Vitamin C (ascorbic acid)	7.72 ± 0.05	7.68 ± 0.04	0.591	7.29 ± 0.05	7.37 ± 0.04	0.282
Other						
Acetone	6.75 ± 0.18	6.61 ± 0.15	0.585	6.20 ± 0.17	5.57 ± 0.14**	0.008
Adenine	6.92 ± 0.12	6.83 ± 0.10	0.610	6.49 ± 0.12	6.05 ± 0.09**	0.008
Ethanol	7.35 ± 0.14	7.30 ± 0.11	0.802	6.83 ± 0.13	6.29 ± 0.10**	0.003
Hypoxanthine	6.76 ± 0.13	6.67 ± 0.10	0.582	6.28 ± 0.13	5.83 ± 0.11*	0.015
Uridine	6.96 ± 0.07	7.05 ± 0.06	0.374	6.63 ± 0.07	6.35 ± 0.06**	0.007

Data are presented as mean ± standard error of mean.

1 Metabolite contents are present in log10 (signal intensity of respective metabolite and arbitrary unit).

* =p < 0.05,

** =p < 0.01,

*** =p < 0.001 compared with single litter size using general linear model procedure. TS=Total solids, SNF=Solids not fat, GPC=Glycerophosphocholine, PC=Phosphocholine, triox=Trimethylamine N-oxide, UDP=Uridine diphosphate

**Figure-2:**
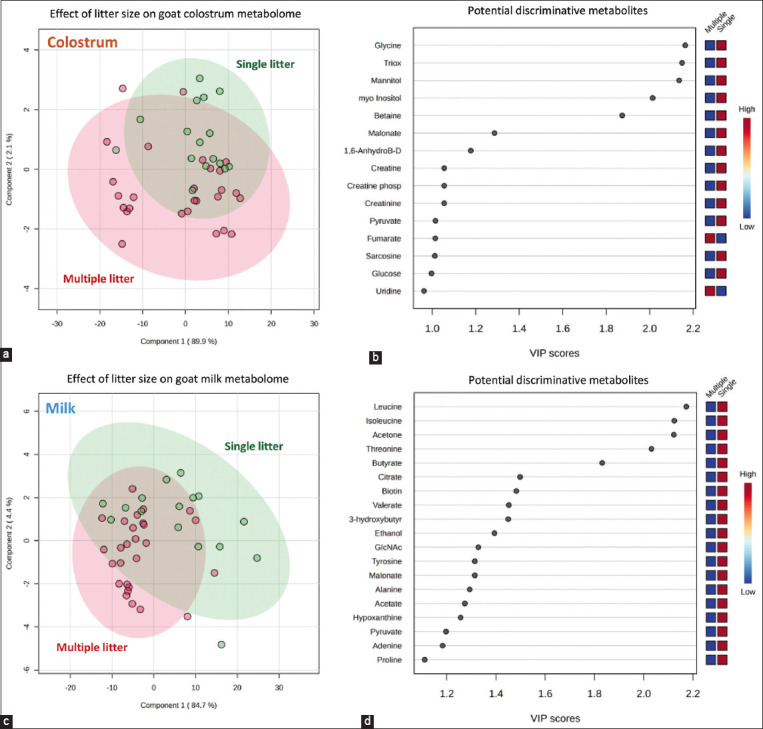
Partial least square discriminant analysis score plot (panel a and c) and variable importance in projection (VIP) scores (panel b and d) reveal the impact of single (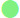) and multiple (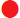) litter size on non-volatile metabolite profiles of colostrum (panel a and b) and mature milk (panel c and d) samples collected from purebred Black Bengal goats. Squares in the VIP score panel express normalized non-volatile polar metabolite content respected to the color range. The red color indicates higher content of the corresponding compound. For interpretation of the references to color in this figure, the reader is referred to the web version of this article and the relative quantification of metabolites in Table-3.

**Figure-3:**
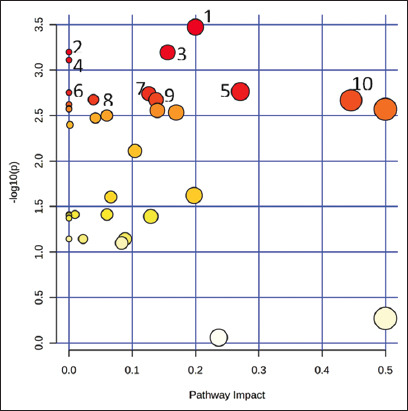
Alterations in the metabolic pathways of single and multiple litter Black Bengal goats (*C. hircus*). The X-axis indicates the pathway impact, and Y-axis indicates the pathway enrichment. Bubbles with darker color and larger size represent higher p-value from enrichment analysis and greater impact from the pathway topology analysis, respectively. Metabolic pathway: (1) biotin metabolism, (2) valine, leucine, and isoleucine degradation, (3) amino sugar and nucleotide sugar metabolism, (4) valine, leucine, and isoleucine biosynthesis, (5) pyruvate metabolism, (6) butanoate metabolism, (7) glycolysis/gluconeogenesis, (8) purine metabolism, (9) glyoxylate and dicarboxylate metabolism, and (10) glycine, serine, and threonine metabolism.

To evaluate the impact of litter size on the goat mammary secretion metabolome, two separate PLS-DA analyses were performed to compare samples collected on the same postpartum day (Figure-2). Results revealed a good separation between single and multiple litter dam samples. A PLS-DA score plot was constructed for the colostrum samples with a prediction accuracy of 69.76%, *R*^2^ = 0.535, and *Q*^2^ = 0.159 (Figure-2a). Considering metabolites with VIP scores >1.0 (Figure-2b) combined with their p-values (Table-3), significant decreases in the concentrations of glycine, triox, mannitol, myo-inositol, and betaine (p < 0.05) were found to be associated with the colostrum derived from goats with multiple litter sizes. In the case of mature milk, another PLS-DA score plot was constructed with a prediction accuracy of 76.74%, *R*^2^ = 0.528, and *Q*^2^ = 0.162 (Figure-2c). VIP scores with a value >1.0 (Figure-2d) and p < 0.05 (Table-3) demonstrated that most indicative metabolites accountable for the discrimination, especially compounds in the group of amino acids and organic acids, were significantly decreased in milk from goats with multiple litter sizes. *In silico* projections using pathway analysis suggested that these significant compounds were derived from the top 10 pathways in *Capra hircus*, which could be differentially regulated in animals with single and multiple litter sizes, as demonstrated in Figure-3.

### Effect of parity number on the major chemical composition and metabolite profiles of colostrum and milk

Colostrum concentrations of fat and TS from the multiparous parity dams were significantly lower than those from the primiparous parity (p < 0.05) (Table-4). However, the parity number of dams had no effect on milk composition.

**Table-4:** Effects of parity number on major chemical composition and non-volatile polar metabolites identified in colostrum and milk of black Bengal goats.

Parameters	Colostrum		p-value	Milk		p-value
	
	Primiparous (n = 18)	Multiparous (n = 25)		Multiparous (n = 25)	Multiparous (n = 25)	
Chemical compositions, %						
Fat (g/100 g)	10.38 ± 0.70	7.78 ± 0.66*	0.013	6.72 ± 0.38	6.87 ± 0.36	0.779
Protein (g/100 g)	16.25 ± 1.06	14.64 ± 1.00	0.288	4.34 ± 0.30	4.77 ± 0.28	0.322
Lactose (g/100 g)	2.98 ± 0.17	3.09 ± 0.16	0.638	4.64 ± 0.08	4.47 ± 0.08	0.154
TS (%)	30.75 ± 1.23	27.01 ± 1.16*	0.039	17.06 ± 0.48	17.44 ± 0.45	0.585
SNF (%)	21.12 ± 0.97	19.96 ± 0.91	0.407	10.35 ± 0.24	10.59 ± 0.23	0.496
Metabolomic profiles^1^ Amino acids and derivatives						
Alanine	7.39 ± 0.15	6.97 ± 0.14	0.055	6.47 ± 0.12	6.46 ± 0.12	0.962
Betaine	8.00 ± 0.04	8.03 ± 0.04	0.610	8.19 ± 0.03	8.19 ± 0.03	0.968
Creatine	7.33 ± 0.09	7.22 ± 0.04	0.395	6.87 ± 0.07	6.92 ± 0.07	0.621
Creatine phosphate	7.33 ± 0.09	7.22 ± 0.04	0.395	6.87 ± 0.07	6.92 ± 0.07	0.621
Creatinine	7.33 ± 0.09	7.22 ± 0.04	0.395	7.23 ± 0.06	7.23 ± 0.06	0.956
Glycine	8.14 ± 0.04	8.14 ± 0.04	0.890	8.31 ± 0.04	8.31 ± 0.03	0.971
Isoleucine	8.14 ± 0.17	7.66 ± 0.16	0.052	7.03 ± 0.17	6.94 ± 0.17	0.713
Leucine	8.02 ± 0.17	7.54 ± 0.16	0.052	6.87 ± 0.18	6.78 ± 0.17	0.721
Methionine	7.37 ± 0.12	7.07 ± 0.11	0.076	6.72 ± 0.10	6.70 ± 0.10	0.868
Proline	8.05 ± 0.10	7.81 ± 0.10	0.114	7.30 ± 0.10	7.30 ± 0.09	0.961
Sarcosine	6.77 ± 0.16	6.46 ± 0.15	0.162	6.13 ± 0.11	6.18 ± 0.10	0.739
Threonine	7.53 ± 0.13	7.20 ± 0.13	0.095	6.61 ± 0.13	6.52 ± 0.12	0.615
Tyrosine	8.35 ± 0.05	8.28 ± 0.04	0.301	7.83 ± 0.04	7.83 ± 0.04	0.983
Valine	7.91 ± 0.16	7.50 ± 0.15	0.073	6.72 ± 0.16	6.67 ± 0.15	0.850
Organic acids and derivatives						
β-hydroxybutyrate	7.85 ± 0.13	7.49 ± 0.13	0.066	6.93 ± 0.11	6.90 ± 0.11	0.859
Acetate	7.17 ± 0.15	6.76 ± 0.14	0.062	6.01 ± 0.12	6.05 ± 0.11	0.811
Butyrate	8.19 ± 0.16	7.73 ± 0.15	0.053	7.29 ± 0.14	7.21 ± 0.14	0.671
Citrate	7.94 ± 0.07	7.98 ± 0.06	0.667	7.84 ± 0.05	7.91 ± 0.05	0.394
Fumarate	6.59 ± 0.07	6.48 ± 0.06	0.235	6.01 ± 0.08	6.02 ± 0.08	0.956
Hippurate	7.16 ± 0.07	7.02 ± 0.06	0.154	6.63 ± 0.07	6.62 ± 0.06	0.944
Lactate	7.53 ± 0.13	7.20 ± 0.13	0.095	6.61 ± 0.13	6.52 ± 0.12	0.615
Malonate	6.58 ± 0.11	6.36 ± 0.10	0.160	6.29 ± 0.06	6.34 ± 0.06	0.605
Pyruvate	7.12 ± 0.06	7.03 ± 0.06	0.283	6.46 ± 0.12	6.50 ± 0.11	0.822
Succinate	7.30 ± 0.15	6.90 ± 0.14	0.063	6.23 ± 0.09	6.25 ± 0.09	0.893
Threonate	6.88 ± 0.15	6.51 ± 0.14	0.078	6.81 ± 0.05	6.83 ± 0.05	0.887
Valerate	8.22 ± 0.16	7.77 ± 0.15	0.055	7.33 ± 0.14	7.25 ± 0.13	0.693
Carbohydrates and derivatives						
Levoglucosan	7.98 ± 0.07	7.88 ± 0.06	0.318	8.06 ± 0.04	8.02 ± 0.04	0.433
Galactose	7.04 ± 0.06	7.02 ± 0.05	0.753	6.67 ± 0.07	6.63 ± 0.07	0.691
Glucose	7.60 ± 0.05	7.52 ± 0.05	0.264	7.35 ± 0.04	7.40 ± 0.04	0.440
Mannitol	8.78 ± 0.04	8.78 ± 0.04	0.965	8.94 ± 0.03	8.49 ± 0.03	0.982
Myo-Inositol	8.23 ± 0.04	8.23 ± 0.03	0.973	6.78 ± 0.05	6.78 ± 0.05	0.945
Acetylglucosamine	7.91 ± 0.11	7.62 ± 0.10	0.061	7.09 ± 0.10	7.05 ± 0.09	0.824
UDP-galactose	7.42 ± 0.06	7.45 ± 0.05	0.744	7.41 ± 0.05	7.40 ± 0.05	0.905
UDP-glucose	7.18 ± 0.06	7.24 ± 0.06	0.529	7.58 ± 0.06	7.57 ± 0.05	0.972
UDP-acetylglucosamine	7.60 ± 0.06	7.61 ± 0.05	0.949	7.24 ± 0.05	7.23 ± 0.05	0.844
Lipids and derivatives						
Acetylcarnitine	7.71 ± 0.07	7.58 ± 0.07	0.190	7.26 ± 0.07	7.25 ± 0.07	0.895
Carnitine	7.69 ± 0.05	7.62 ± 0.05	0.345	7.46 ± 0.04	7.48 ± 0.04	0.779
Choline	7.42 ± 0.05	7.36 ± 0.05	0.490	7.09 ± 0.06	7.08 ± 0.5	0.894
GPC	8.35 ± 0.05	8.28 ± 0.05	0.312	7.82 ± 0.04	7.82 ± 0.04	0.977
PC	8.30 ± 0.05	8.22 ± 0.05	0.283	7.73 ± 0.04	7.74 ± 0.04	0.873
Triox	8.09 ± 0.04	8.11 ± 0.04	0.627	8.26 ± 0.03	8.25 ± 0.03	0.934
Vitamins						
Vitamin B2 (riboflavin)	7.42 ± 0.07	7.43 ± 0.06	0.920	7.24 ± 0.08	7.20 ± 0.07	0.714
Vitamin B5 (pantothenate)	8.18 ± 0.18	7.66 ± 0.17*	0.045	7.09 ± 0.18	6.99 ± 0.17	0.681
Vitamin B7 (biotin)	7.91 ± 0.15	7.49 ± 0.14	0.061	6.85 ± 0.15	6.83 ± 0.14	0.925
Vitamin C (ascorbic acid)	7.72 ± 0.05	7.69 ± 0.05	0.639	7.36 ± 0.05	7.30 ± 0.05	0.453
Others						
Acetone	6.90 ± 0.17	6.46 ± 0.16	0.071	5.83 ± 0.16	5.95 ± 0.15	0.595
Adenine	6.99 ± 0.11	6.76 ± 0.11	0.146	6.32 ± 0.11	6.22 ± 0.10	0.545
Ethanol	7.51 ± 0.13	7.15 ± 0.12	0.059	6.58 ± 0.12	6.54 ± 0.11	0.840
Hypoxanthine	6.83 ± 0.12	6.60 ± 0.11	0.192	6.13 ± 0.12	5.98 ± 0.11	0.407
Uridine	7.03 ± 0.06	6.98 ± 0.06	0.605	6.49 ± 0.07	6.49 ± 0.06	0.934

Data are presented as mean ± standard error of mean.

1 Metabolite contents are present in log10 (signal intensity of respective metabolite and arbitrary unit). *=p < 0.05 compared with multiparous using general linear model procedure. TS=Total solids, SNF=Solids not fat, GPC=Glycerophosphocholine, PC=Phosphocholine, triox=Trimethylamine N-oxide, UDP=Uridine diphosphate

**Figure-4:**
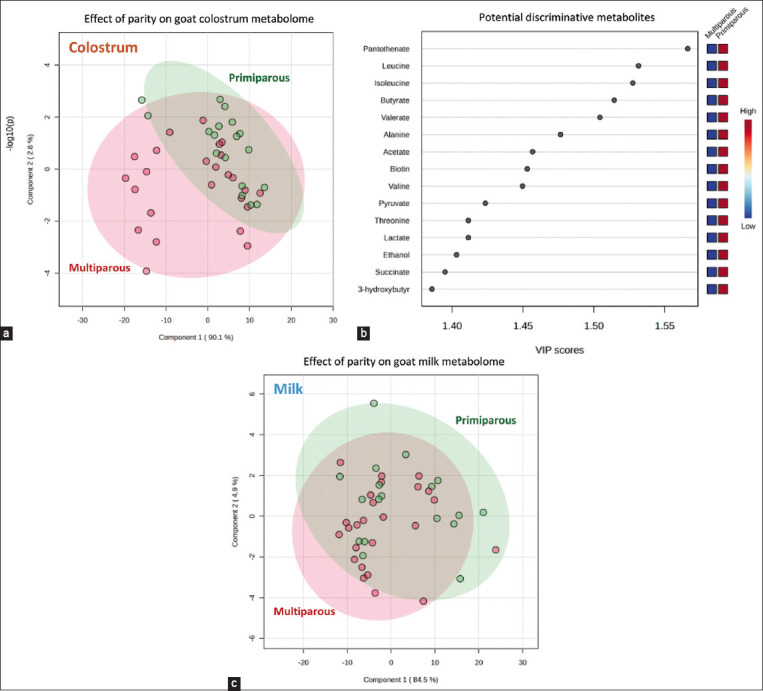
Partial least square discriminant analysis score plot (panel a and c) and variable importance in projection (VIP) scores (panel b) reveal the impact of animal parity number, that is, primiparous (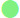) and multiparous (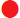), on non-volatile metabolite profiles of colostrum (panel a and b) and mature milk (panel c) samples collected from purebred Black Bengal goats. Squares in the VIP score panel express normalized non-volatile polar metabolite content respected to the color range. The red color indicates higher content of the corresponding compound. For interpretation of the references to color in this figure, the reader is referred to the web version of this article and the relative quantification of metabolites in Table-4.

To evaluate the impact of parity number on the goat mammary secretion metabolome, two separate PLS-DA analyses were performed to compare samples collected on the same postpartum day (Figure-4). A good separation between primiparous and multiparous goats was only observed in colostrum samples, as revealed by a PLS-DA score plot with a prediction accuracy of 65.11%, *R*^2^ = 0.546, and *Q*^2^ = 0.325 (Figure-4a). VIP scores with a value >1.0 (Figure-4b) and p < 0.05 (Table-4) revealed only a significant difference in the concentration of pantothenate or Vitamin B5 (p = 0.045) between the two groups of samples. It should be mentioned that variations in the concentrations of leucine, isoleucine, butyrate, valerate, and alanine in goat colostrum appeared to be affected by the parity number of animals, with p-values ranging from 0.052 to 0.055 (Table-4). In contradiction with colostrum, it was remarkable that the goat parity number negligibly affected variation in the metabolite profiles of mature milk. The PLS-DA score plot with a prediction accuracy of 58.14%, *R*^2^ = 0.417, and *Q*^2^ = 0.150 (Figure-4c) demonstrated no distinguishing pattern between the metabolite profiles of milk from primiparous and multiparous goats. This result corresponds well with no statistically significant level of metabolites found between the two groups of samples in Table-4.

## Discussion

This study included goats aged <1 year to 5.8 years old, with parity numbers ranging from 1 to 7. Most dams give birth to both twins and singletons. The litter size from this study was 1.79, which is slightly lower than the number found in 2019 (1.9 ± 0.1) [6].

^1^H-NMR analysis identified fifty-one distinct metabolites in this study, surpassing previous reports. In the study of bovines, there were previously identified 45 metabolites [17] and 28 metabolites [11] using the same technique. In goat and bovine mammary secretions, chemical shifts displayed comparable values. The metabolomic profile of goat colostrum is examined for the 1^st^ time in this study, while a prior investigation on goat milk powder consisting of 44 metabolites has been demonstrated [9]. The results may have differed due to the use of milk powder instead of raw milk in their study. Milk powder production through heat processing may result in the destruction of water-soluble vitamins. The higher metabolite count in this study could be a result of differences in species, preparation methods, and identification techniques.

### Differences in the major chemicals and metabolomic profiles between colostrum and milk

Starting on day 7, the transition from colostrum to mature milk led to decreased fat and protein concentrations, lowering TS and SNF, but increasing lactose concentration. The previous study by Romero *et al*. [5] and Buranakarl *et al*. [6] yielded a comparable result. The protein and fat contents of colostrum are significantly higher than those in mature milk produced after day 5 [18]. Milk lactose percentage and yield rose in cattle from colostrum to 3 months postpartum [19]. As the primary driver of osmolarity in mammary gland secretions, lactose draws in more water to enhance milk production within mammary epithelial cells [20].

The heat map’s separated colors indicate that most amino acid, organic acid, and lipid contents were reduced in the transition from colostrum to milk for metabolomic profiles. The present study’s findings concurred with those of earlier research on bovine colostrum [11, 17]. Amino acids are milk protein components, mainly casein [21]. The elevated amino acid and protein levels in colostrum indicate significant amino acid availability during pregnancy for *de novo* protein synthesis. The transition to milk of the study’s samples might account for their high betaine and glycine content, which could be attributed to their role as organic osmolytes and contributing factors to superior milk production post-delivery [22].

Organic acids were more concentrated in colostrum than in milk. β-hydroxybutyrate and acetate participate in milk fat synthesis in mammary epithelial cells [23], contrastingly, other organic acids function as gluconeogenesis precursors [24]. Butyrate from dam rumen fermentation is linked to milk fat production in the mammary gland [25]. During pregnancy, the dam exhibited a heightened metabolic process involving glycolysis, lipolysis, and gluconeogenesis, necessitating large quantities of lactate, citrate, pyruvate, and malonate. To ensure dam health and colostrum production, the dam’s metabolism will be accelerated during the peripartum period.

Most carbohydrates and derivatives, excluding lactose and mannitol, were more concentrated in colostrum than in milk. In infancy, when the rumen is still immature, the high sugar content in colostrum may serve as an essential energy source. Lactose and mannitol serve distinct functions. The lactose content in milk is greater than in colostrum due to their roles as organic osmolytes. Milk yield was shown to be positively linked to lactose content [20]. As lactose concentration rose, the levels of its precursors, glucose, and uridine diphosphate-galactose, diminished. Prolactin may facilitate increased lactose synthesis during the transition from colostrum to milk. β1,4-galactosyltransferase and α-lactalbumin expression levels were increased following prolactin administration [26]. Transitioning colostrum to milk with added betaine, glycine, mannitol, triox, and lactose results in a higher yield but a dilution effect that lowers concentrations of other milk components.

The metabolites in the lipids and derivatives group were predominantly higher in colostrum. Acetylcarnitine and carnitine facilitate fatty acid metabolism and oxidation. Milk phospholipids are predominantly produced in mammary epithelial cells through *de novo* synthesis [27]. Products of blood phosphatidylcholine breakdown, glycerophosphocholine and fatty acids, can be utilized to synthesize milk triglycerides. The milk glycerophosphocholine to phosphocholine ratio serves as a prognostic biomarker for ketosis risk [28]. Colostrum contains more water-soluble vitamins than milk. These vitamins (B2, B5, B7, and C) serve as cofactors that boost the metabolic processing of nutrients, particularly macromolecules, within animals [29–31]. After a few days of birth, a kid’s gastrointestinal tract may absorb higher quantities of vitamins, impacting their development. Vitamins B5 and B7 are essential for synthesizing coenzyme A and for hair, nail, and skin strengthening in neonates [32]. In addition, ascorbate is known as a potential factor for kid performance and health status, as seen in calves [33].

Acetone, adenine, hypoxanthine, and uridine were slightly lower in milk, which might be due to the dilution effect. The level of acetone in the present study was presumed to be normal, whereas a higher level was associated with ketosis [34].

### Effect of litter size on the metabolomic profiles

#### Colostrum

Multiple dams yielded a greater amount of colostrum fat and TS. Their unaltered condition was previously reported regardless of litter size [5, 6]. Low-molecular-weight metabolites in BB goat colostrum were unaffected by litter size. In multiple litter sizes, the contents of betaine, glycine, mannitol, myoinositol, and triox were significantly lower compared to single litter sizes, making these compounds potential differentiators for litter size. Unlike other metabolites, the major osmolyte, lactose in colostrum, remained unchanged. Further investigation is needed to determine the impact of dam litter size on colostrum yield.

#### Milk

In providing milk to multiple offspring, dams exhibit statistically lower levels of fat, protein, TS, and SNF, along with a higher lactose concentration. About 60% or more of the identified metabolites showed lower levels in milk from multiple litter size goats compared to single litter size goats, as evidenced by a PLS-DA score plot. According to the VIP score, leucine, isoleucine, acetone, threonine, and butyrate were identified as the potential discriminant metabolites for litter size in milk. Despite litter size influencing most metabolites, its impact on their metabolic pathways might not be directly implied. Higher lactose with lower most of metabolites in milk of dams with multiple litter size may suggest higher milk yield reported by Dhara *et al*. [35]. The higher the number of fetuses during pregnancy, the greater the placental lactogen levels in goats [36]. In ewes, higher milk yield in twin litter size was also found to be positively associated with the concentration of growth hormone [37].

### Effect of the parity number on metabolomic profiles

#### Colostrum

The fat and TS levels in colostrum from multiparous dams were significantly lower than those from primiparous dams, but protein, lactose, and SNF remained unchanged. The fat content of goat colostrum from multiparous dams was comparable to that reported earlier [5]. In the metabolomic profile, Vitamin B5 was found to be the distinguishing factor with lower levels in the colostrum of multiparous dams compared to primiparous dams as indicated by the VIP score. Vitamin B5 is essential to metabolism due to its incorporation into coenzyme A and acyl carrier protein [38]. Vitamin B5 is derived from forage and produced by rumen microorganisms. This study reveals that colostrum has a greater amount of Vitamin B5 than milk, potentially contributing to newborn growth and development. In the present study, Vitamin B5 levels were higher in the colostrum of primiparous dams than in multiparous dams and may be related to lipid biosynthesis, resulting in higher fat content because supplementation of Vitamin B5 in cows increases milk fat [39]. The aging process might decrease the amount of Vitamin B5 in an animal’s blood and colostrum due to impaired absorption/utilization or altered microbial activity of gut micro-organisms.

#### Milk

Milk’s composition and metabolites remained unchanged. The fat content of goat milk remained unchanged as parity number increased, as reported by Zamuner *et al*. [40]. Likewise, a similar pattern emerged in sow milk [12]. The parity number had a negligible impact on mammary secretion.

## Conclusion

^1^H-NMR analysis of colostrum and milk from BB goats identified 51 metabolites in their mammary secretions. Except for lactose, betaine, glycine, mannitol, and triox, major and minor milk compounds concentrations were lower than those in colostrum. This implies a higher milk yield from the dilution effect. In multiple litter size dams, milk exhibited higher lactose and lower other metabolites compared to milk from single litter size dams due to increased milk yield for multiple kids. The dam’s parity did not impact the milk metabolomic profile. The significance of specific metabolites in metabolomic profiles for kid’s growth requires further investigation. This study has a limitation. In some dams, colostrum samples were not collected immediately after birth due to nighttime deliveries in this study. Samples were collected within 3 h of delivery.

## Authors’ Contributions

TTPV, CB, SC, and MN: Data curation. TTPV, CB, MN, and SS: Formal analysis. TTPV, CB, ST, SC, and PR: Methodology. CB: Project administration. TTPV and CB: Original draft. CB, SC, and SS: Supervision and writing – review and editing. All authors have read, reviewed, and approved the final manuscript.
